# Environmental pollution and its impact on hypertension: a review

**DOI:** 10.3389/fpubh.2025.1637703

**Published:** 2025-09-30

**Authors:** Li Che, Zilong Wang

**Affiliations:** ^1^Department of Cardiology, Central Hospital of Dalian University of Technology, Dalian, China; ^2^Department of Neurosurgery, The First People's Hospital of JinZhou District, Dalian, China

**Keywords:** environmental pollution, hypertension, cardiovascular diseases, biological mechanisms, public health

## Abstract

This review aims to summarize the impact of environmental pollution on the development of hypertension. The processes of urbanization and industrialization have significantly increased various types of pollution in our surroundings. Research indicates that exposure to these pollutants can negatively affect cardiovascular function, which is a recognized risk factor for both the onset and progression of hypertension. In this review, we systematically summarize current findings, explore the relationship between environmental pollution and hypertension, discuss potential biological mechanisms underlying this association, and suggest directions for future research. Understanding these relationships is crucial for developing public health strategies aimed at preventing heart disease caused by pollution.

## 1 Introduction

Hypertension is a common chronic condition that significantly impacts global health (1). Numerous environmental factors have been linked to the onset and progression of hypertension, with growing attention on how environmental pollutants contribute to this issue. Airborne pollutants, including fine particulate matter (PM2.5), heavy metals, and volatile organic compounds (VOCs), have been associated with hypertension. These pollutants enter the body primarily through inhalation and skin contact, triggering inflammatory responses and causing vascular damage, which can lead to increased blood pressure (BP). For instance, PM2.5 is known to induce oxidative stress, which is critical in the development of hypertension. Additionally, heavy metals like lead (Pb) and cadmium (Cd) disrupt normal cardiovascular function, while VOCs can affect hormonal regulation of BP. Understanding the pathways that connect these pollutants to hypertension is essential for developing effective strategies to reduce their impact on cardiovascular health (2–4). This review aims to enhance our understanding of how these pollutants affect hypertension and to inspire new research in this important field.

Air pollution, particularly in the form of PM2.5, has emerged as a significant factor contributing to hypertension. These tiny particles penetrate deep into the lungs, where they instigate local oxidative stress and inflammation. This response in the lungs triggers the release of inflammatory substances into the bloodstream, which can lead to various cardiovascular issues (5). Research indicates that short-term exposure to elevated levels of PM2.5 is linked to increased BP and a rise in hospitalization rates due to hypertension. For example, one study found that a 10 μg/m^3^ increase in PM2.5 concentration was associated with a notable increase in both systolic and diastolic BP among individuals with hypertension (6). The impact of PM2.5 on BP is particularly significant in older adults and those with other health conditions (7). Mechanistically, exposure to PM2.5 is connected to oxidative stress and inflammation, all of which are closely related to the development and progression of hypertension (8).

Heavy metals, including Pb, Cd, and mercury, are significant environmental pollutants that have been linked to hypertension. Recent research indicates that exposure to these metals can disrupt various physiological functions, leading to an increase in BP. In this regard, a co-relational study has reported an increased level of heavy metal in hair and nails of hypertensive patients, which correlated with its presence with dyslipidemia and oxidative stress status (9). Potential mechanisms are induction of inflammatory pathways and disturbance of endothelial function, leading to vascular injury and eventually hypertension (3). Concentration of multi heavy metal might be one of the factors to increase the risk of hypertension, so it is necessary to take environmental assessment including heavy metal for the people with high BP (10).

VOCs are a primary group of environmental pollutants associated with hypertension. New research finds that some VOCS can be associated with increased BP. For example, urinary metabolites of certain VOCs are correlated with both systolic and diastolic BP in studies that include nonsmokers (11). Given the omnipresence of VOC in urban areas, it is crucial to understand their role in contributing to hypertension as a basis for public health intervention (11).

Further study is needed to determine the precise pathophysiologic mechanisms of how environmental pollutants are linked with hypertension. Longitudinal studies designed to investigate the cumulative effects of multiple pollutant exposures on BP and cardiovascular health over extended periods of time are needed. Moreover, investigations might need to investigate influences of the environment on genetic susceptibility for hypertension, and subsequently examine preventive measures targeting those risks. The growing global epidemic of hypertension must be combated through implementation of effective public health interventions to reduce exposure to environmental chemicals. Incorporating environmental health information into cardiovascular health interventions is an important approach that may lead to improved health outcomes for at-risk populations (12).

## 2 Types of environmental pollutants and their sources

### 2.1 Fine particulate matter and its characteristics

PM particularly PM2. 5, is a harmful air pollutant composed of fine particles with a diameter less than 2.5 micrometers. It is critical to note that PM2.5 is not a homogenous pollutant. Its chemical composition (e.g., sulfate, nitrate, organic carbon, black carbon, heavy metals) and subsequent health effects vary significantly depending on the emission source, such as fossil fuel combustion from automobile traffic, industrial processes, or biomass burning from residential heating or wildfires (13). Their small size allows them to be deposited deep in the alveolar region of the lungs, where they can induce oxidative stress and inflammation (8). PM2. 5 composition data varies by location and season, and is typically made up of a combination of organic and inorganic substances. This wide variance makes the impact of it on health difficult to gage (6). PM2. 5 are significantly affected by meteorological parameters, urbanization factor, and legislation. Those with relatively stringent environmental regulation have low PM2. 5, at least in the area of policy creation in a belt air pollution control management (14). Possible reasons of climatic season differences in PM2. 5 is anyone heating their homes more in the winter, or more traffic in the summer. This supports the need for continued monitoring to help identify key interventions to lessen exposure to these toxicants (15, 16).

### 2.2 Biological pollutants and their sources

Biological pollutants refer to pathogenic microorganisms, allergens, and biological toxins that threaten human cardiovascular health. They mainly come from damp indoor spaces, outdoor greenery, and polluted biological waste. Exposure to bacterial endotoxins may induce chronic inflammation-activating pro-inflammatory cytokines [e.g., tumor necrosis factor-alpha (TNF-*α*), interleukin-6 (IL-6)] and increasing Reactive Oxygen Species (ROS) production (17).

### 2.3 Heavy metals and their exposure pathways

Toxic heavy metals as Pb and Cd represent, in fact, meaningful health hazards, particularly in urban environment. Various pathways can introduce these metals to the human body, such as via inhalation, ingestion, and dermal contact. Pb exposure frequently occurs in older housing stock, especially in older urban centers where lead-based paint and plumbing are still used. The health effects of heavy metals are of great concern especially in vulnerable populations, as children and developing fetus are susceptible to developmental delays and cognitive impairments caused by Pb exposure (18). Chronic kidney disease (CKD) and some bone disorders have been linked with long-term exposure to Cd, emphasizing the importance of robust monitoring and remediation programs in Cd-contaminated regions (19, 20). Public health interventions should focus on strategies to reduce heavy metal exposure, such as stricter regulations, public awareness campaigns, and more aggressive clean-up efforts. Strategies aimed at mitigating the negative health impacts of heavy metals include stricter control of emissions from industrial sources and safer disposal of waste. Additionally, public awareness campaigns can educate individuals about the dangers of lead exposure and ways to reduce it. Implementing remediation programs that detoxify contaminated soil and water supplies is also crucial in lessening the adverse health effects on human populations. Together, these actions contribute to fostering a healthier environment and decreasing the prevalence of diseases induced by heavy metals (21).

### 2.4 VOCs and their sources

VOCs are a group of organic chemicals that readily evaporate at typical room temperatures, leading to air pollution and contributing to the formation of ground-level ozone. These compounds are commonly released into the environment through automotive exhaust, industrial activities, and the use of paints and solvents in consumer products (22–24). In urban settings, VOC emissions are particularly linked to transportation, especially during peak traffic hours when congestion is at its highest. The risks associated with air pollution are notably heightened in densely populated areas, where a significant number of vehicles produce considerable amounts of airborne pollutants. This relationship between traffic density and VOC emissions highlights the urgent need for targeted measures to improve air quality in urban environments (25). A large amount of VOCs are emitted to the atmosphere from various industrial processes, such as the manufacture of petrochemicals and biomass burning, leading to deteriorated air quality (26, 27). Health effects can range from mild to severe after exposure to VOCs. Symptoms include eye, nose and throat irritation, but more severe health effects are also possible, from respiratory effects that may compromise breathing and lung function to neurological side effects that may harm cognition and brain health (28, 29).

Furthermore, certain VOCs are recognized as precursors to secondary pollutants, such as ozone and PM. This relationship complicates air quality management strategies because these compounds can undergo chemical reactions that lead to the formation of harmful pollutants, adversely affecting both the environment and human health. Therefore, it is essential to understand the role of VOCs in air pollution, as this knowledge is crucial for managing their impact—not only at the point of initial release but also regarding the pollutants generated through subsequent chemical reactions, which contribute to poor air quality (30). To address this issue, various strategies can be employed, including stricter emission controls, the use of low-VOC products, and increasing public awareness about the sources of VOCs and their associated health risks (26).

### 2.5 Physical pollutants and their sources

Radiofrequency (RF) radiation (including 5G, WiFi and mobile communication) has evolved from a negligible pollutant three decades ago to a widespread pollutant now with substantially increased levels of daily exposure to humans. RF radiation interferes with mitochondrial function and induces the activation of Nicotinamide Adenine Dinucleotide Phosphateoxidase in vascular cells, resulting in overproduction of ROS, decreasing Nitric Oxide (NO)—an important vasodilator levels, increasing vascular resistance (31).

### 2.6 Interactions among environmental pollutants

For chemicals, PM2.5 can adsorb VOCs to form more toxic complexes that are capable of inducing vascular inflammation (32). For biologicals, pollen and dust mites may synergize to worsen allergic reactions, indirectly worsening pollution-induced hypertension (33). RF radiation increases the permeability of vascular cell membranes, allowing more PM2.5-bound heavy metals to enter cells and further aggravate oxidative stress.

## 3 Environmental pollution and hypertension epidemiological studies

### 3.1 Overview of large-scale population studies

In recent years, a number of population-based epidemiological studies have provided strong evidence of a significant association between environmental pollution and hypertension. A related study investigated National Health Service data in Scotland, and demonstrated a strong association between air pollution including especially particulate matter exposures (PM10 and PM2. 5) and Nitrogen Dioxide (NO2) and hypertension. In this analysis, the relation between these pollutants and the number of cases of hypertension among a cohort of 776,579 patients with a diagnosis of hypertension was assessed by means of multivariate regression models (34).

The findings confirmed that air quality and traffic noise significantly predict the prevalence of hypertension, highlighting the importance of considering environmental factors in relation to this health issue. The CHCN-BTH study, which included 32,135 participants, revealed that long-term exposure to ambient air pollution and traffic-related pollution notably heightened the risk of developing hypertension, especially under stricter diagnostic criteria (6). These results underscore the necessity for public health interventions at the population level that focus on mitigating environmental exposures to help reduce the risk of hypertension.

### 3.2 Regional study results analysis

Geo-epidemiology focuses on the important connections between household and air pollution and their impact on hypertension, examining geographic and demographic factors throughout the region. For instance, a time-series analysis in Lanzhou found that daily NO2 and carbon monoxide (CO) were positively associated with hospitalization rate for hypertension. The association was predominately observed during winter when pollution levels were elevated, which was suggestive of potential seasonal variation in the health effects of air pollutants among susceptible populations (7). A recent study in the Niger Delta region of Nigeria reported a disturbing rate of CKD and systemic hypertension in communities highly exposed to air pollutants (35). This finding has immediate implications for cardiovascular health and the need to tackle environmental exposure, interventions that may have profound impact on those exposed, particularly vulnerable persons. This subnational assessment highlights the significance of specific public health policy to local environmental risk and health outcomes.

### 3.3 Special studies on women and the older adult(s)

Studies in susceptible populations, such as women and the older adult(s), indicate that they are more likely to develop hypertension related to environmental pollution. Meta-analysis indicates that women exhibit a tendency to develop hypertension more readily than men when exposed to air pollution (36). This difference may reflect physiologic and life style differences which exist between both sexes. Women’s vulnerability to pollution-induced hypertension is governed by sex hormones, primarily menopause: pre-menopausal women have high levels of estrogen, a robust vascular endothelial NO synthesis capacity and high anti-inflammatory activity that shields them from the toxicity of pollutants such as PM2.5, demonstrating a lower incidence of hypertension than age-matched men; post-menopausal ovarian insufficiency leads to a steep decline in estrogen, the protective effect is lost and susceptibility to pollutants increases dramatically (37, 38). In addition, menopausal women often present with autonomic disorders and enhanced hypothalamic–pituitary–adrenal (HPA) axis activity (39). The older adult(s), and the older adult(s) with chronic medical issues, are particularly affected by the health hazards of pollution, according to studies (40). Patients with hypertension who acquire Coronavirus Disease (COVID) have substantially higher rates of acute kidney injury and death, for example. This phenomenon is rooted in an elevated risk of external exposure to poor environmental quality that can exacerbate their health condition and lead to more severe outcomes (41). The results highlight the importance of tailored interventions that address the unique health needs of women and older adults to facilitate an appropriate public health response to the specific environmental health vulnerabilities faced by these populations.

## 4 The biological mechanisms by which environmental pollution affects hypertension

Pollution has emerged as a significant contributor to high BP, with various physiological pathways linking the two. Understanding these pathways is crucial for developing targeted interventions to mitigate the health risks posed by environmental contaminants.

### 4.1 Inflammatory response and oxidative stress

Indeed, chronic exposure to some environmental pollutants—air pollution being the most extensively studied group—has been associated with increased inflammation and oxidative stress, which are key pathogenic hallmarks for the development of hypertension. Particles such as particulate matter (PM2.5 and PM10), NO2, and sulfur dioxide (SO2) can induce the generation of ROS and pro-inflammatory cytokines. These contribute to disorders such as vascular inflammation, which play a central role in the pathogenesis of high BP (42). For instance, the inflammatory response elicited by simultaneously activated immune cells followed by the release of cytokines (TNF-*α*, IL-6) as mediators of the development of vascular inflammation and hypertension (8). Oxidative stress also affects the availability of NO required for vasodilation. A reduced NO response translates into higher vascular resistance and consequently higher BP (43). This establishes a vicious cycle whereby inflammation leads to oxidative stress and oxidative stress exacerbates inflammation, both sustaining hypertension (44).

### 4.2 The role of the autonomic nervous system

Role of the autonomic nervous system (ANS) in cardiovascular effects of environmental pollutants: Environmental pollutants in the cross hairs of the ANS. These pollutants induce a shift in the sympathovagal balance in the presence of these provoking factors. These disturbances lead to an increase in sympathetic tone that has been linked to elevates heart rate and BP (45). The mechanistic chain begins with inhalation of pollutants such as PM2.5: these pollutants first stimulate pulmonary inflammatory receptors, triggering the body’s anti-inflammatory reflex—a protective mechanism that relies on parasympathetic activation to suppress excessive inflammation. However, when pollutant exposure intensity exceeds the body’s compensatory capacity, parasympathetic function becomes suppressed: this not only disables the anti-inflammatory reflex (leading to chronic activation of inflammatory responses) but also disturbs core ANS balance, shifting it toward sympathetic predominance (46).

### 4.3 The mediating role of cortisol

Environmental pollutants (PM2.5, VOCs) may influence the level of cortisol through the activation of the HPA axis. Short term exposure to environmental pollutants could activate the HPA axis to secrete cortisol, which would then regulate BP by modulating sodium and water excretion. Long term or repeated exposure to pollutants would cause dysfunction of the HPA axis with abnormal rhythms of cortisol secretion, such as loss of the circadian rhythm and elevated basal levels (47).

### 4.4 The impact on kidney function

Renal function is a key determinant of BP control and is harmed by environmental pollution. Pollutants may cause oxidative stress and inflammation in the kidneys that results in nephron injury and CKD (35). Air pollution exposure has also been correlated with greater urinary excretion of biomarkers of kidney injury, such as β2-microglobulin (48). Running this correlation suggests that even short-term exposure to these chemicals has short-term adverse effects on kidney function. The kidneys regulate BP mainly through the renin-angiotensin-aldosterone system (RAAS). Renal function is regulated by RAAS and pollution-induced renal dysfunction can upset this system triggering high BP (49).

Hypertensive crises should be a significant concern for patients with CKD, as hypertension is closely linked to this condition. Recent studies have identified environmental pollution as a new risk factor that may accelerate the progression of CKD. This relationship complicates the existing understanding of how pollution affects BP; exposure to various pollutants can not only lead to a decline in kidney function but also exacerbate hypertension, creating a harmful cycle for the kidneys (35). It is crucial to manage hypertension effectively. The kidneys, which filter blood, are vulnerable to environmental pollutants such as heavy metals, particulate matter, and chemicals. These substances can disrupt kidney function, leading to changes in BP and ultimately resulting in hypertension. With this knowledge, public health initiatives and individual lifestyle choices can be adjusted to mitigate the harmful effects of these pollutants on health. By recognizing the environmental factors that influence kidney health, clinicians can provide better guidance to their patients on hypertension management and develop tailored prevention strategies that address both personal and community health needs.

## 5 Importance of government policies on environmental pollution and cases

### 5.1 Impact of government policies on environmental pollution control

Actions at the level of individual governments can strongly influence the management of environmental pollutants. Health impacts and air pollution have been demonstrated to be mitigated through carefully designed regulation. One of the starkest examples is the suite of collective prevention and control measures that have been undertaken in China; reductions in PM2.5 in border areas have been observed, with a 3.5% reduction when implemented (50). The Two Control Zones policy was associated with improved public health outcomes, reducing respiratory illness by 5.7%. Gains in public health have been achieved through tightened environmental standards (51). Local officials’ commitment to environmental governance can therefore increase the effectiveness of such policies, which in turn create the spillover effects that prompt neighboring regions to do likewise (52). There is abundant evidence that a strong government structure is critical to implementing environmentally protective reforms that curtail pollution and safeguard public health.

### 5.2 Case analysis of community intervention measures

Such initiatives can significantly promote sustainable lifestyles and enhance awareness among citizens. For example, the implementation of air environmental audits in various cities across China has shown promising results. These audits have led to a notable decrease in emissions of air pollutants, which correlates with better adherence to air quality regulations, highlighting how our communities are making strides in improving air quality and environmental health (52). Additionally, the River Chief System has had a remarkable effect by substantially reducing industrial wastewater emissions through the encouragement of enterprise accountability (53).

Such measures are correlated with better compliance with environmental standards and more proactive citizen involvement in pollution control. These interventions frequently tailor solutions to address the local environmental challenges communities encounter, and are therefore more likely to be contextually appropriate and implementable; hence incorporating local knowledge and resources could lead to greater effectiveness and rebuttal of the notion that local adaptation is an excuse (51). Community involvement is an integral part of environmental control and protection of public health.

### 5.3 Importance of awareness raising and public education

Awareness and educating the public—two critical elements of effective environmental health interventions can capture attention. The public’s perception of the risks of environmental pollution has been demonstrated to have a substantial impact on adherence to health advice and the motivation to act proactively to avoid causing any harm (54, 55). Health awareness of air quality has infiltrated the home after mass campaigns, thereby increasing awareness of the consequences of individual behaviors. The growing awareness of pollution issues has led many individuals to feel a stronger commitment to pollution control (56, 57). In this context, educational initiatives should aim to bridge knowledge gaps, particularly among vulnerable groups who may struggle to access vital information (58). The role of media is essential in effectively communicating this information and reaching various segments of society, whether through mass media, audiovisual platforms, or social media (59). Education efforts should not only focus on fostering individual responsibility but also on nurturing a culture of accountability, empowering communities to advocate for cleaner environments and push for impactful changes in public health. To achieve the long-term behavioral changes necessary for effective environmental health policies, it is crucial to invest in comprehensive educational campaigns.

This review primarily focuses on chemical pollutants; however, it is important to recognize that the range of environmental threats to cardiovascular health also encompasses biological and physical pollutants. Among these, anthropogenic electromagnetic fields (EMF), particularly RF radiation (RF-EMR) emitted by wireless technologies, represent a widespread and emerging physical pollutant. Emerging evidence suggests that RF-EMR exposure may elicit oxidative stress. Investigation of the independent and combined effects of these multiple environmental insults (chemical, biological, physical) on the development of hypertension is warranted. Such knowledge will be critical to developing strategies to safeguard public health from the multitude of insults encountered in our environment.

## 6 Advancing research on emerging pollutants: interactions, multifactorial analysis, and individual susceptibility in hypertension development

### 6.1 Research needs for emerging pollutants

The persistent detection of emerging pollutants such as pharmaceuticals, personal care products, and microplastics poses significant risks to human health and the environment. Recent research underscores the necessity for comprehensive studies on the sources, hazards, and control methods of these contaminants. Among the potential solutions, biochar has emerged as a promising option due to its demonstrated effectiveness adsorbent and as a method to address the problem of water pollution (60). Additionally, the role of climate change on emerging pollutants is an issue that requires further investigation since it significantly influences the environmental behavior and ecotoxicity of these contaminants (61). Rather than concentrating on single pollutant studies, which have long been prevalent, future studies should target multi-pollutant interactions and synergistic effects to gain a holistic understanding of their ecological and human health implications. This necessitates interdisciplinary collaboration between environmental scientists, toxicologists and public health professionals (62). Furthermore, there is a pressing need for novel strategies and technologies to mitigate and monitor the impacts of anthropogenic pollutants to ensure that regulatory frameworks keep pace with the ever-evolving landscape of environmental contaminants (63–65).

### 6.2 Interaction effects and multifactorial analysis

The interplay between pathogenic factors from the environment and the amplification of their effects on different diseases such as hypertension remains to be elucidated. There is already evidence from previous studies that both air pollution and traffic noise, two pervasive characteristics of urban settings, independently contribute to increased risk of hypertension; however, the overall extent of their combined effects remains unknown (49). Future studies should employ multivariate approaches to quantify how these environmental stressors may interact and amplify cardiovascular risks. For instance, concurrent exposure to particulate matter and noise pollution has been associated with substantial elevations in BP. It is possible that simultaneous exposure has a synergistic effect, which will not be detectable when each element is tested individually (66). This allows for uncovering complex associations between multiple environmental exposures and health outcomes by integrating epidemiological data with robust statistical models. This approach will facilitate the development of more effective public health interventions and policies aimed at alleviating the burden of hypertension in vulnerable populations (8). By employing a multi-faceted strategy, we can gain deeper insights into the biological mechanisms that drive these interactions, thereby enabling the creation of targeted preventive measures.

### 6.3 Individual susceptibility and hypertension development

Understanding how specific risk factors contribute to the development of hypertension is crucial for the identification of high-risk groups and the design of effective prevention strategies. Hypertension is a multifactorial disease involving genetic background, lifestyle factors, and environmental exposures. Recently, it was shown that genetic polymorphism, e.g., of the CYP2C19 gene, can affect an individual’s predisposition to hypertension (67). Moreover, the interplay between genetic susceptibility and environmental factors, such as exposure to heavy metals and air pollutants, adds another layer of complexity to the pathogenesis of hypertension (29). Future studies should focus on elucidating these interactions, ideally in diverse populations, to pinpoint high-risk subgroups that might benefit from early intervention and personalized treatment. Moreover, investigating the impact of psychosocial factors (e.g., stress and financial situation) on the risk of hypertension would provide a more comprehensive picture of individual susceptibility (68). This combines genetic, environmental and psychosocial information to develop better screening and treatment approaches. These interventions are either designed to lower risk of hypertension in high-risk populations or intended to improve public health features more broadly.

## 7 Conclusion

Environmental pollution may cause hypertension, and its complex relationship has received increasing attention in the last few years, which parallels an emerging consensus among healthcare professionals about the complexity of this public health crisis. Epidemiologic studies have revealed a strong association between exposure to diverse pollutants and the development of hypertension. Additionally, a number of biological mechanisms underlying this association have now been identified, emphasizing the necessity of integrating research perspectives to fully understand the possible impact of environmental toxins on cardiovascular disease.

From a research perspective, however, it is crucial to integrate the diverse research outcomes, which typically stem from different research methodologies, study populations, and ecological settings. For instance, some studies concentrate on particulate matter and its cardiovascular effects, whereas others indicate emerging pollutants like microplastics and endocrine disruptors. This heterogeneity presents challenges and opportunities for future study. Developing this interdisciplinary intersection between epidemiologists, toxicologists, and clinicians provides a broader framework for understanding how these pollutants interact with various susceptibility factors, including genetics and lifestyle.

Needless to say, when exploring the effects of emerging pollutants, we should consider population-based susceptibility. Differences in genetic susceptibility, socioeconomic status, and comorbidities can significantly modify the impact of environmental pollutants on hypertension. Additional population-specific research will clarify the effect of the population-level impact of COVID and help to translate research into bespoke public health interventions tailored to those most affected.

The insights derived from this line of research, at the end of the day, hold significant implications for public health policy. Regulation of pollutants, particularly those characterized as major determinants of hypertension, should be a top priority for policymakers, along with investing in the research of novel solutions to reduce exposure. Increase public awareness about environmental pollution risks so people can choose healthy lifestyles and make efforts to reduce pollution.

Overall, although the interaction between environmental pollution and hypertension is complex, it can be elucidated utilizing collaborative and multi-dimensional approaches. Bridging the divides between research perspectives and shifting attention to the implications of emerging pollutants may see more pieces in the puzzle fitting together to inform public health efforts. These strategies will reduce harassment behaviors of hypertension and enhance cardiovascular health in all populations.
